# Unraveling Dual Cognitive Disorders: A Case Report and Literature Review on Marchiafava–Bignami Disease and Possible Alzheimer’s Disease

**DOI:** 10.3390/diseases13090310

**Published:** 2025-09-22

**Authors:** Floris Petru Iliuta, Mirela Manea, Aliss Madalina Mares, Corina Ioana Varlam, Constantin Alexandru Ciobanu, Adela Magdalena Ciobanu, Radu-Mihail Lacau, Mihnea Costin Manea

**Affiliations:** 1 Department of Psychiatry and Psychology, Discipline of Psychiatry, Faculty of Dental Medicine, “Carol Davila” University of Medicine and Pharmacy, 010221 Bucharest, Romania; floris.iliuta@umfcd.ro (F.P.I.); mirela.manea@umfcd.ro (M.M.); aliss-madalina.mares@drd.umfcd.ro (A.M.M.); radu-mihail.lacau@drd.umfcd.ro (R.-M.L.); mihnea.manea@umfcd.ro (M.C.M.); 2 Department of Psychiatry, “Prof. Dr. Alexandru Obregia” Clinical Hospital of Psychiatry, 041914 Bucharest, Romania; adela.ciobanu@umfcd.ro; 3 Faculty of Medicine, “Carol Davila” University of Medicine and Pharmacy, 010221 Bucharest, Romania; alexandru.ciobanu2020@stud.umfcd.ro; 4 Neurosciences Department, Discipline of Psychiatry, Faculty of Medicine, “Carol Davila” University of Medicine and Pharmacy, 020021 Bucharest, Romania

**Keywords:** Alzheimer’s disease, Marchiafava–Bignami disease, neurodegenerative disorders, young-onset dementia, chronic alcohol consumption

## Abstract

Alzheimer’s disease (AD) is the most prevalent form of dementia, particularly in those aged 65 and older. Dementia can also occur under age 45, known as young-onset dementia (YOD), although this is rarer. Marchiafava–Bignami disease (MBD) is a rare disorder characterized by demyelination and necrosis of the corpus callosum, primarily affecting individuals with chronic alcohol use. We present the case of a 49-year-old woman admitted for psychiatric and neurological evaluation due to a multidomain cognitive disorder with a sudden onset approximately four years prior, which progressed rapidly, resulting in complete dependence on others for daily activities. Her medical history included moderate depression, chronic alcohol consumption, and professional exhaustion. Psychological assessments revealed severe neurocognitive impairment. MRI scans highlighted significant bilateral parietal atrophy, hippocampal atrophy, and demyelinating lesions in the corpus callosum, consistent with MBD. Despite initial inconsistencies in biomarkers, later tests showed elevated tau protein, phosphorylated tau, and amyloid-beta, supporting an AD diagnosis. Clinical presentation, combined with neuroimaging findings and chronic alcohol consumption history, led to a diagnosis of AD with young onset and chronic MBD. This case illustrates the complexities involved in diagnosing overlapping neurodegenerative disorders. The coexistence of MBD and AD complicates the treatment plan, requiring a multifaceted approach addressing both neurodegenerative and nutritional aspects.

## 1. Introduction

Dementia describes a significant decline in cognitive ability that interferes with daily activities. Alzheimer’s disease (AD) is the most prevalent form of dementia, but it can also appear before age 65, known as early- or young-onset dementia (YOD). AD is a neurodegenerative condition with a gradual onset and progressive cognitive and behavioral impairment. It typically appears after age 65 (late-onset AD or LOAD), while early-onset AD (EOAD) occurs before 65, affecting about 5% of AD patients and often presenting atypical symptoms, leading to delayed diagnosis and a more aggressive course [1,2]. It can even occur under the age of 45, where it is known as young-onset dementia (YOD), which is a form of dementia diagnosed earlier in life and includes both neurodegenerative and reversible causes [3,4].

AD’s neurodegenerative process begins in the entorhinal cortex within the hippocampus, with genetic factors contributing to both early- and late-onset AD. Risk factors include advancing age, depression, cardiovascular and cerebrovascular disease, smoking, family history of dementia, chronic alcohol use, and the APOE ε4 allele [5]. In the case of YOD, besides the primary cause, which is AD, other reversible causes include inflammatory, infectious, toxic, and metabolic etiologies, which are not unique to YOD and can also complicate late-onset dementia [6]. Although these features are relevant for differential diagnosis, they were not all demonstrated in our patient.

Significant progress has been made in developing biomarkers for early and specific diagnoses of AD. These include neuroimaging markers from amyloid and tau PET scans, cerebrospinal fluid (CSF) markers, and plasma markers like amyloid-beta 42 (Aβ42), phosphorylated tau (p-tau), and total tau (t-tau) [7].

Genetic testing for AD is not routinely recommended but may be considered for rare early-onset cases. The APOE ε4 allele is a significant risk factor, with heterozygous carriers having a threefold increased risk and homozygous carriers a fifteenfold increased risk of developing AD. The ε4 allele’s presence is crucial for sporadic AD, though it does not guarantee disease development [5,8].

Routine lab tests like complete blood count, comprehensive metabolic panel, thyroid-stimulating hormone, and vitamin B12 levels help exclude other cognitive impairment causes, but they do not reveal specific AD abnormalities [9]. A brain CT may show cerebral atrophy and a widened third ventricle, but this is not specific to AD. An MRI is superior for evaluating dementia, revealing atrophy in the entorhinal cortex and medial temporal cortex, and particularly the hippocampus, which is characteristic of AD and associated with memory decline. However, hippocampal atrophy also occurs in normal aging, making the role of volumetric MRI in early AD detection and differentiation from normal aging still debatable and under research [9,10,11].

Chronic alcohol consumption is a significant risk factor for various neurological disorders, including young-onset dementia (YOD), Wernicke–Korsakoff Syndrome, Marchiafava–Bignami disease, hepatic encephalopathy, cerebellar degeneration, and peripheral neuropathy.

Marchiafava–Bignami disease (MBD) is a very rare disorder characterized by demyelination and necrosis of the corpus callosum and adjacent subcortical white matter, predominantly affecting malnourished patients with alcohol use disorder [12]. The rarity of diagnosed cases of MBD, which progress to full-blown syndrome with high lethality, explains its low incidence. However, Castaigne (1971) and Lechevalier (1977) reported cases surviving 5–10 years after diagnosis, with a markedly thinned corpus callosum at autopsy [13,14].

MBD mainly affects middle-aged men, but cases have also been described in women [15,16]. The disease can be acute, subacute, or chronic, with symptoms including dementia, dysarthria, spasticity, and walking inability, and patients may enter into a coma or a demented state for years, with outcomes ranging from spontaneous recovery to death [17,18].

Originally diagnosed only at autopsy, advances in MRI now allow for accurate in vivo diagnosis. Lesions can appear as hypodense regions in the corpus callosum on tomography and as areas with diminished T1 signal and increased T2 signal on MRI, and an interhemispheric disconnection syndrome has been observed in survivors; patients with alcohol use disorder without hepatic disease, amnesia, or cognitive dysfunction show thinning of the corpus callosum on autopsy and MRI, indicating that alcohol or malnutrition can damage the corpus callosum without causing the necrotic lesions seen in MBD [19,20,21,22].

MBD is most commonly found in malnourished patients with chronic alcohol use disorder [23], especially involving red wine, but also with whisky, rice wine, or liquor, with some cases reported without alcohol consumption, for example, in those with poorly controlled diabetes mellitus [24,25]. The etiology is thought to involve alcohol-induced neurotoxicity and B-complex vitamin deficiencies [26]. Other causes include sudden serum osmolality fluctuations [27], non-alcohol-related malnourishment after gastric bypass surgery [28], and conditions like carbon monoxide poisoning, sepsis, cerebral malaria, sickle cell disease, and cardiac carcinoma surgery [25,29,30,31,32,33]. Although clinical features can be variable and nonspecific, MBD should be suspected in patients with chronic alcohol use and/or malnutrition who present with common neurological and psychiatric symptoms, including psychotic and emotional disorders that may appear acutely, sub-acutely, or chronically [26,34,35]. Acute presentation includes a sudden loss of consciousness, seizures, apathy, aggressiveness, confusion, and psychosis. Subacute features include depression, ataxia, apraxia, agraphia, anomia, dysarthria, and visual dyslexia, potentially as part of an interhemispheric disconnection syndrome. Chronic forms can present as progressive severe global dementia, visual hallucinations, auditory delusions, and behavioral abnormalities, with signs of interhemispheric disconnection syndrome. Another classification pattern based on clinical status and brain injury detectable by magnetic resonance includes two types of presentation.

Type A is characterized by severe consciousness deficit, seizures, dysarthria, and hemiparesis, with hyperintense swelling of the corpus callosum observed on MRI, and associated with a worse prognosis. On the other hand, type B is characterized by dysarthria, gait disturbance, interhemispheric disconnection symptoms, and less impairment in consciousness, with only partial callosal lesions on MRI, and is associated with a better prognosis [34,36].

MRI represents the gold standard method for diagnosing MBD, although CT scans may reveal hypodense lesions in the corpus callosum, particularly in the central portion [37,38]. Pathognomonic MRI features include symmetrical lesions on the corpus callosum, typically restricted to the genus, body, or splenium [39]. In the acute stage, these lesions exhibit cytotoxic edematous changes, with hyperintense signals on T2-weighted/fluid-attenuated inversion recovery (FLAIR) and diffusion-weighted imaging (DWI), often forming a “sandwich sign” in the middle layer of the corpus callosum [37,38,40]. In the post-acute and chronic stages, typical radiological findings include symmetric atrophy of the corpus callosum, with variable focal hyperintensities on T2-weighted images and focal hypointensities on T1-weighted images. These changes reflect regional necrosis and possible cyst formation. Additionally, lesions may be found in other brain regions, indicating a poorer prognosis. As the acute stage progresses, edema resolves, and MRI hyperintensities may normalize [39]. Early diagnosis and treatment may lead to a complete resolution of corpus callosum lesions on MRI. However, untreated patients or those who do not respond to treatment may experience permanent demyelination and necrosis, with the MRI showing thinning, atrophy, and cystic transformation of the corpus callosum [23,39]. Key differential diagnoses include Wernicke encephalopathy, which is also associated with thiamine deficiency but has distinct brain lesions [41]. Epileptic seizures, though a potential symptom of MBD, can also be a primary disorder. Acute encephalitis and stroke can mimic MBD’s symptoms, but imaging and clinical evaluation can help distinguish them. Additionally, other demyelinating diseases affecting the nervous system need to be considered. While there is no specific treatment proven to reverse MBD, addressing potential thiamine deficiencies with intravenous (parenteral) thiamine administration is a common approach [42].

Incomplete lesions with relative sparing of superior commissure fibers are hypothesized to correlate with a better prognosis compared to lesions extending into the convolution white matter. Poor prognosis and/or severe dementia are associated with extra-callosal lesions, impairment of cerebral lobes, severe consciousness disturbance, and heavy alcohol consumption. Early diagnosis and effective treatment are crucial for patient recovery, with serial MRI demonstrating a complete disappearance of lesions in some cases with prompt diagnosis and treatment, which further emphasizes the importance of MRI as a diagnostic and monitoring tool in general psychiatry and neurology, extending beyond degenerative disorders [39,43,44].

This case is unique due to the complexity of diagnosing young-onset dementia with features suggestive of AD and MBD in the same patient. We hypothesize that chronic alcohol-related MBD may have interacted with underlying neurodegenerative processes to accelerate cognitive decline. The present case report integrates a detailed case study with a brief literature review to highlight diagnostic challenges and implications for clinical practice.

### Methods

Neuropsychological assessments (MMSE, MoCA, Hamilton Anxiety Rating Scale, Beck Depression Inventory, Clock Drawing Test, Reisberg scale, and GAF) were performed in a standardized clinical setting by trained psychiatrists under consistent conditions, with normal ranges provided in Table 1. MMSE and MoCA were administered face to face in quiet environments with no time constraints beyond test protocols. The literature review was conducted in PubMed and Scopus (2010–2024) using keywords ‘Marchiafava-Bignami Disease,’ ‘Alzheimer’s disease,’ ‘young-onset dementia,’ and ‘dual pathology,’ with inclusion of case reports, reviews, and original studies relevant to dual cognitive impairment. MRI was performed in T1 and T2/FLAIR sequences (on T1-weighted MRI, demyelinated regions appear hypointense, while on T2/FLAIR sequences they appear hyperintense.

## 2. Case Presentation

We present the case of a 49-year-old female patient who was hospitalized for psychiatric and neurological evaluation in the context of a multidomain cognitive disorder with sudden onset approximately 4 years ago.

The patient presented 4 years ago with a vertiginous syndrome, classified as vestibular neuritis, and completely remitted under specific treatment, without impairment of cognitive status recorded at that time by the neurologist. After 6 months, after a few days of absence from work (affirmatively the first leave in about 10 years), she had an episode of spatial disorientation, subsequently noting a decrease in the skills necessary to perform at work (acalculia, working memory disorders with prolonged time required to perform tasks, and language disorders with elements of anomic aphasia, including in foreign languages in which she was previously fluent). There was no history of fever, headache, vomiting, seizure, or hypertension. It was difficult to precisely identify when the cognitive deficits began, as they were observed progressively over several months. Cognitive decline evolved rapidly, with the patient becoming completely dependent on the family in performing daily activities.

The patient admits chronic consumption of alcohol (1 L of wine/day for about 10 years, affirmatively stopped 4–5 years ago) and professional exhaustion (for 10 years, worked more than 12 h/day without any leave of absence). No smoking or disorders involving other substances except alcohol were present. The patient was diagnosed with moderate depression, right vestibular neuronitis, and congenital myopia, and denied allergies. Hereditary and collateral history revealed four maternal aunts with medically undocumented neurocognitive disorders.

The results of medical check-ups carried out four years ago were within normal limits. Also, the patient underwent a first cerebrospinal fluid (CSF) examination, which revealed tau protein, phosphorylated tau, and beta-amyloid within normal limits, with negative 14-3-3 protein in the CSF. Apolipoprotein E genotyping detected the presence of only the E3 genotype. The E4 allele was not present. The electroencephalogram was normal. Doppler of the cervical cerebral vessels identified hypoplasia of the right vertebral artery with dominant left vertebral artery (anatomical variant), as well as fetal ACP variant on the right side (anatomical variant).

**Figure 1:**
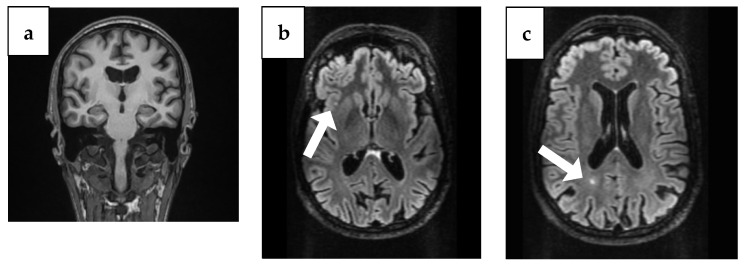
Brain MRI findings from 4 years ago. (**a**) Coronal plane MRI scan illustrating temporal atrophy. (**b**) Arrow: Axial plane MRI scan illustrating a right insular lesion. (**c**) Arrow: Axial plane MRI scan illustrating in the occipital horn of the paraventricular right lateral ventricle.

The brain CT showed bilaterally enlarged pericerebral fluid spaces, accentuated at the parietal level, suggestive for brain atrophy. Cranial MRI showed triventricular hydrocephalus and maxillary and ethmoid and bilateral frontal sinusitis, but the following neurosurgical consultation revealed temporal atrophy and ex vaquo ventriculomegaly without hydrocephalus, which usually occurs when a degenerative disease like AD, stroke, or trauma and causes damage to the brain that may result in shrinking of the brain tissue. Additionally, the MRI identified the presence of two punctiform lesions in FLAIR, under 5 mm in diameter, in the supratentorial subcortical white matter; the first one was on the right insula and the second one, paraventricular, was in the occipital horn of the right lateral ventricle (Figure 1).

Treatment with memantine 20 mg/day and sertraline 100 mg/day was started. There was an additional indication for rivastigmine patches of 9.5 mg/day, which were not administered due to patient non-compliance.

At every annual psychiatric evaluation since the onset of the condition, the patient presented with marked intrapsychic tension, basal anxiety, depressive mood with a tendency toward social withdrawal, anhedonia, apathy, and organically imprinted personality, with symptoms worsening rapidly every year for the last 3 years.

Psychological examinations carried out every year from the onset of the disorder to the present revealed a score of 16/30 on the Mini-Mental State Examination in the first two years, characteristic of a moderate neurocognitive disorder, and in the last year, the score decreased to 9/30, showing a severe neurocognitive disorder requiring permanent care and supervision.

Last year, the result on the Montreal Cognitive Assessment (MoCA) was 4/30 points, revealing severe cognitive impairment. The Hamilton Anxiety Rating Scale highlighted moderate anxiety, with a score of 17/56, and the Beck’s Depression Inventory revealed severe depression, with a score of 32/63.

The results of Sunderland’s clock drawing test three years ago were initially a score of 7/10, and then in the second year 6/10, and at the last examination it had dropped to 1/10. In the first two years, the Reisberg scale had a score of 5/7, representing a moderate to severe cognitive decline, and then at the last evaluation, the score was 6/7, more precisely representing severe cognitive decline. The Global Assessment of Functioning scale in the first year at the first presentation showed a score of 30/100, in the second year 20/100, and finally at the last presentation showed a score of 10/100.

Psychometric evaluations were performed by certified psychologists. Although the MMSE and MoCA revealed severe decline, interpretation must be cautious as depressive symptoms may have negatively impacted test performance.

At the last psychiatric evaluation, carried out this year, the patient presented hypobulia, apathy, hypersomnia, lack of emotions, no capacity for self-care, requiring permanent assistance, and a high risk of wandering and injury, which was especially important because patients in permanent need of supervision often need more invasive psychiatric procedures such as electroconvulsive therapy [45].

General and neurological clinical examinations were within normal limits. The examination of cortical function revealed temporal disorientation, partial spatial orientation, fixation and evocation hypomnesia, acalculia, slight elements of anomia, execution of simple and inconstantly complex commands, inconstant left–right disorientation, agnosia, digital agnosia, and adermolexia, with emotional regression and tendency to infantilization, with all these symptoms concluding in a multidimensional cognitive disorder. The patient never exhibited epileptic seizures during follow-ups.

The electrocardiogram and electroencephalogram were within normal parameters.

The blood count, ionogram, coagulogram, and vitamin D and B levels were within normal laboratory-approved limits, and the infectious disease panel (HIV, syphilis, hepatic viruses B and C, Borrelia Burgdorferi) showed no signs of an external pathogen-related cognitive disorder. Immunogram, urinalysis, and bacterial urine tests were within normal limits. Anticardiolipin antibodies and anti-β2 glycoprotein-1 antibodies, NMDA antibodies, and antinuclear antibodies were within normal limits, with a positive lupus anticoagulant test in isolation but without other autoantibodies or clinical features of systemic lupus erythematosus; thus, a diagnosis of lupus was excluded.

The second examination of the cerebrospinal fluid, performed 3 years after first one, was macroscopically normal, with normocytemia, proteinuria and glycoprotein within limits, but revealed elevation of tau protein (877 pg/mL), phosphorylated tau (63.6 pg/mL) and amyloid-beta 42 (0.94) (Table 2), with negative antineuronal antibody blot as follows: amphiphysin 1, CV2 (CRMP5)-abs, PNMA2, Ri ag-abs (ANNA type II), Yo-abs (Purkinje cells), Hu D ag-abs (ANNA type I), and negative electrophoresis of serum proteins with immunofixation. Tau PET was not performed.

The second brain-contrast MRI, which was performed after 3 years, did not reveal recently ischemic or hemorrhagic lesions but identified the same two punctiform hyperintense lesions in FLAIR under 5 mm in diameter in the supratentorial subcortical white matter; the first one was on the right insula and the second one paraventricular in the occipital horn of the right lateral ventricle, representing demyelinating lesions. Brain imaging also showed cortical and subcortical atrophy associated with hippocampal atrophy, with the Medial Temporal Lobe Atrophy (MTA or Scheltens) scale rated on coronal T1-weighted images scoring 2, which means a widening of choroid fissure and of the temporal horn of the lateral ventricle. This score is determined through visual assessment of the width of the choroid fissure, the width of the temporal horn, and the height of the hippocampal formation. A score of 0 indicates no atrophy, 1 indicates only widening of the choroid fissure, 2 includes widening of the temporal horn of the lateral ventricle, 3 denotes moderate hippocampal volume loss (decrease in height), and 4 indicates severe hippocampal volume loss. In individuals under 75 years, a score of 2 or more is considered abnormal, whereas in those over 75, a score of 3 or more is abnormal. The MTA score is considered the best marker of hippocampal atrophy, with an MTA ≥ 1.5 cut-off recommended for AD diagnosis in individuals under 75 years [46]. The MRI also showed significant bilateral symmetric parietal atrophy, with a Koedam score representing the posterior atrophy score of parietal atrophy of grade 2, meaning substantial sulcal widening with substantial gyral atrophy.

**Figure 2:**
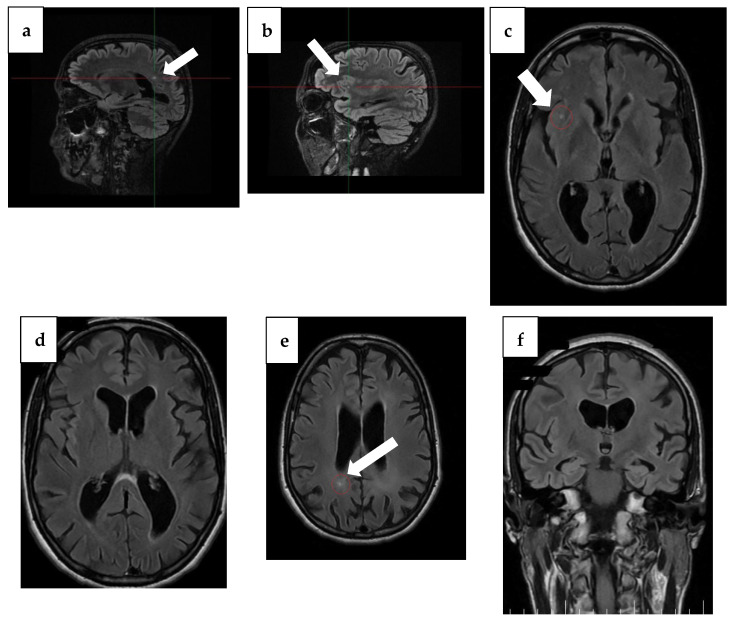
MRI findings from 1 year ago. (**a**) Sagittal plane MRI scan illustrating the lesion (arrow) in the occipital horn of the paraventricular right lateral ventricle. (**b**) Sagittal plane MRI scan illustrating a right insular lesion (arrow), (**c**) axial plane MRI scan illustrating a right insular lesion (arrow), (**d**) axial plane MRI scan illustrating a lesion in the occipital horn of the paraventricular right lateral ventricle, (**e**) Arrow: axial plane MRI scan illustrating hyperintensity on T2/FLAIR and hypointensity on T1 in the splenium of the corpus callosum, suggestive of demyelination in the context of MBD, (**f**) coronal plane MRI scan illustrating global atrophy with cortical, subcortical, and hippocampal atrophy.

**Figure 3:**
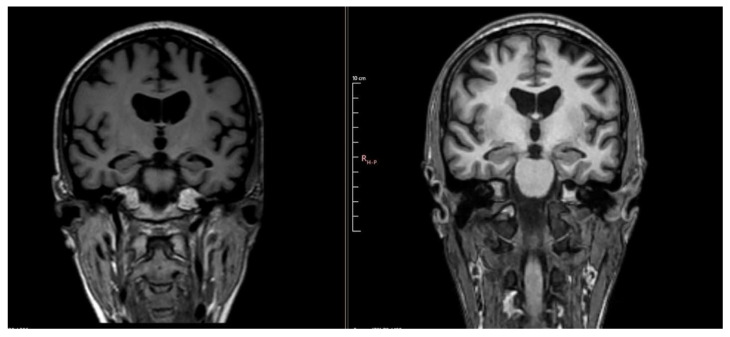
Comparative images showing progression of atrophy between MRI 4 years ago (**left**) and MRI 1 year ago (**right**).

Notable changes were found, with progression of atrophy compared to the previous examination from 3 years ago and severe atrophy of the corpus callosum, resulting in marked thinning on MRI. In addition to this, T2/FLAIR hyperintensity in the splenium of the corpus callosum with T1 hypointensity was detected, which may be suggestive of corpus callosum demyelination in the context of MBD (Figure 2 and Figure 3).

Hippocampal and parietal atrophy raised the suspicion of AD, although these changes are not entirely specific. Hippocampal atrophy may also occur in major depressive disorder, where structural imaging studies have consistently demonstrated reduced hippocampal volumes compared to controls [47]. Similarly, volumetric MRI studies show that hippocampal and entorhinal atrophy can be observed in alcohol-related brain damage and in MBD, complicating the differential diagnosis [48]. Therefore, the imaging findings in our patient must be interpreted with caution, as they cannot by themselves establish a definitive diagnosis of AD. Thus, the sandwich sign, pathognomonic for MBD, can no longer be seen at the time of the second MRI, with the thinning of the corpus callosum being too pronounced. This maybe could have been noted at an earlier stage, when atrophy of the corpus callosum would have been less.

The diagnosis of AD with young onset and chronic MBD (closer to type B) was established based on family history; the long-standing prior history of alcohol consumption; psychometric and clinical evidence of marked global cognitive impairment; neurological examination; and characteristic MRI findings of global cerebral, hippocampal, and corpus callosum atrophy.

Treatment consisted of the administration of thiamine, with no favorable response, and antidementia drugs—the association of memantine at 20 mg/day with donepezil with a gradual increase up to the maximum dose of 10 mg/day without benefit, alongside the increase in the dose of SSRI antidepressant sertraline to 150 mg/day with limited improvement. Vitamin therapy at home with thiamine and cyanocobalamin was combined with this regimen; a lack of thiamine response and absence of mammillary body involvement argued against Wernicke–Korsakoff encephalopathy. Also, general lifestyle changes were advised: recommendations were for a diet rich in fruits, vegetables, greens, and fish, with hypolipidic and hypoglycidic elements, and aerobic physical activity, within tolerability, for 30–60 min daily, as well as social activity and the initiation of individual psychotherapy and systemic psychotherapy with the family.

Table 3 summarizes key results of investigations chronologically, including year of evaluation, MMSE/MoCA, CSF tau/p-tau/Aβ42, MRI findings (parietal, hippocampal, callosal changes) and clinical summary.

## 3. Discussion

We presented a case of a patient with multidomain cognitive disorder with initial non-amnestic onset, progressive evolution, and significant worsening of deficits compared to previous evaluations, with brain imaging showing important biparietal atrophy, also evolving compared to the previous investigation. These aspects are suggestive of neurodegenerative pathology, most likely Alzheimer’s disease. However, we noted great variability and multiple inconsistencies in the patient’s cognitive deficits during hospitalization, and the absence of associated biomarkers of Alzheimer’s disease (tau protein, phosphorylated tau, and beta-amyloid) in corticospinal fluid at the first dosing two years ago. However, the second evaluation after two years revealed elevated tau protein (877 pg/mL) and phosphorylated tau (63.6 pg/mL) and elevated amyloid-beta 42 (ratio 0.94). The AD diagnosis was considered probable, not definite, based on this later elevation of tau, p-tau, and elevated amyloid-beta 42 associated with hippocampal/parietal atrophy. We acknowledge that these findings are not definitive.

Thus, investigative work-up at readmission after 3 years included another lumbar puncture and fluid analysis to exclude infectious/paraneoplastic/autoimmune pathologies that could explain the decline in cognitive functions. There are no arguments for autoimmune encephalitis, with a lack of pleocytosis, EEG within normal limits, and no brain lesions suggestive of this; neuroinfection screening and anti-neuronal antibodies were negative. Also, there are no arguments for prion disease based on neurological examination, EEG, and brain imaging at more than 4 years after onset. At the same time, we note the lack of arguments for another metabolic disease. In the same context, given the imaging appearance suggestive of corpus callosum severe atrophy, we also considered a possible superposition with MBD. The possibility that the patient’s severe depression–anxiety syndrome may interfere with cognitive function testing cannot be excluded. In order to clarify the etiology, it would be useful to evaluate preferably with FDG-PET or SPECT and genetic testing for dementia, which were not available to us.

The differentiation between MBD from AD is difficult to make, with several clinical and radiological considerations required. The presence of specific lesions in the corpus callosum, seen in the MRI, suggests MBD, especially with a history of alcohol use, while generalized atrophy of the brain and the presence of associated biomarkers of AD (tau protein, phosphorylated tau, and beta-amyloid) indicate AD. The challenges in diagnosis between MBD and AD lie in their overlapping clinical and imaging features, though the underlying causes differ. Both conditions can present with cognitive decline, memory impairment, confusion, and changes in behavior and personality. While MBD is linked to chronic alcohol consumption and demyelination of the corpus callosum, AD involves neurodegenerative processes such as amyloid plaque buildup and neurofibrillary tangles. It is also important to take into account that depression and chronic alcohol use may have contributed to both cognitive decline and neuroanatomical findings.

In some cases, particularly in patients with a history of chronic alcohol use, symptoms of MBD, like confusion, speech disturbances, and ataxia, may be the first signs. These can progress into cognitive decline and memory issues more characteristic of AD. MRI scans might show lesions in the corpus callosum associated with MBD, along with generalized atrophy in the hippocampus and cerebral cortex, which are typical of AD. In the case of our 49-year-old patient, with chronic alcohol use and a family history of neurocognitive disorders (including four affected aunts), the rapid cognitive decline over three years, with a sudden onset, requires a thorough investigation to distinguish between possible diagnoses. The swift progression and sudden onset of symptoms, combined with her history of alcohol consumption, strongly point towards MBD. This is typically characterized by symmetric atrophy of the corpus callosum with focal hyperintensities on T2-weighted MRI and hypointensities on T1-weighted MRI. However, given her family history, AD must also be considered, as it is usually associated with generalized atrophy of the hippocampus and cortex.

The case suggests that the patient may have initially presented with symptoms of MBD, as indicated by the MRI findings and clinical presentation, and later developed symptoms consistent with AD. This is supported by the progression to imaging findings like hippocampal and cortical atrophy, typical of AD. This scenario raises the hypothesis that MBD, possibly exacerbated by chronic alcoholism, preceded or co-occurred with the development of AD.

The clinical process described in the case relates to the pathophysiology of both MBD and AD, albeit through different mechanisms. The symptoms initially suggestive of MBD, such as memory issues and behavioral changes, can be linked to the corpus callosum demyelination typical of MBD, which disrupts communication between the hemispheres of the brain. This demyelination is usually a result of chronic alcohol consumption, leading to direct toxic effects and nutritional deficiencies, particularly in B vitamins like thiamine. As the clinical course progressed, features more typical of AD emerged, including more pronounced memory loss, language difficulties, and evidence of cortical atrophy on imaging. These symptoms align with the pathophysiological hallmarks of AD, such as amyloid plaque accumulation and neurofibrillary tangles, which lead to neuronal death and brain atrophy. Hippocampal atrophy is nonspecific and may result from depression, aging, or alcohol-related damage. Although biomarker abnormalities suggest possible AD pathology, these findings are not definitive. MBD, chronic alcohol use, and depression provide the most consistent explanation for this case. The convergence of these factors highlights the need for a detailed clinical evaluation and comprehensive imaging studies to ascertain the extent of overlap between these conditions and to guide a multifaceted treatment approach.

## 4. Conclusions

This case illustrates the complexity of diagnosing dementia in the context of chronic alcohol use, psychiatric comorbidity, and overlapping imaging and biomarker findings. The clinical picture is most consistent with chronic Marchiafava–Bignami disease, with possible but unconfirmed superimposed Alzheimer’s disease suggested by later biomarker changes. Depression and anxiety likely contributed to both symptom expression and neuropsychological test performance. Rather than demonstrating a proven overlap between AD and MBD, this case underscores the need for caution in interpretation, the importance of longitudinal follow-up, and the value of advanced imaging and genetic evaluation in resolving diagnostic uncertainty. Future research should include the longitudinal follow-up of patients with suspected dual pathology, advanced imaging modalities such as positron emission tomography (PET), and systematic investigations into genetic and metabolic contributors. These may improve diagnostic accuracy and therapeutic approaches for patients with overlapping neurodegenerative conditions. This case underscores the diagnostic challenges of YOD, where MBD, alcohol-related neurotoxicity, and psychiatric comorbidities may mimic AD. Our findings highlight the importance of multimodal evaluation and caution against over-interpreting nonspecific imaging or biomarker abnormalities as AD.

## Figures and Tables

**Table 1:** Neuropsychological Assessments in the Patient with Reference Ranges.

Assessment	Patient Score (Year 0)	Patient Score (Year 3)	Reference Range (Normal)
**Mini-Mental State Examination (MMSE)**	16/30	9/30	≥27
**Montreal Cognitive Assessment (MoCA)**	-	4/30	≥26
**Hamilton Anxiety Rating Scale (HAM-A)**	29	34	Mild < 17; Moderate 18–24; Severe ≥ 25
**Beck Depression Inventory (BDI)**	32	29	Normal < 13; Moderate 14–28; Severe ≥ 29
**Clock Drawing Test (Sunderland scale)**	7/10	5/10	≥9/10
**Reisberg Global Deterioration Scale (GDS)**	3	6	1 = No impairment; 7 = Severe impairment
**Global Assessment of Functioning (GAF)**	65	30	91–100 = Healthy; ≤20 = Severe impairment

**Table 2:** Cerebrospinal fluid (CSF) biomarker results with reference ranges.

Biomarker	Baseline (Year 0)	Year 3	Reference Range (Normal)
**Tau (pg/mL)**	Normal	↑ 877 pg/ml	<450 pg/ml
**Phosphorylated Tau (p-Tau181, pg/mL)**	Normal	↑ 63.6 pg/ml	<61 pg/ml
**Amyloid-beta 42 (Aβ42, ratio)**	Normal	0.94 (↓)	≥1.0
**14-3-3 Protein**	Negative	-	Negative

↑: above reference range, ↓: below reference range.

**Table 3:** Summarization of key results of investigations in chronological order.

Year/Timepoint	MMSE/MoCA	CSF Biomarkers	MRI Findings	Clinical Course
Baseline (4 years ago)	MMSE 16/30	Tau, p-Tau, Aβ42 within normal limits; 14-3-3 negative	Temporal atrophy; two punctiform FLAIR lesions (insula + occipital horn); ex vacuo ventriculomegaly	Initial cognitive decline after vestibular neuritis; onset of disorientation and working memory/language deficits
Year 1 (3 years ago)	MMSE 16/30	Not repeated	Progressive cortical/subcortical atrophy; lesions unchanged	Dependence increasing, moderate neurocognitive disorder
Year 2 (2 years ago)	MMSE 12/30	Not repeated	Parietal atrophy becoming evident	Rapid decline, moderate-to-severe impairment
Year 3 (1 year ago)	MMSE 9/30, MoCA 4/30	Tau ↑ 877 pg/mL; p-Tau ↑ 63.6 pg/mL; Aβ42 ↓ 0.94	Hippocampal atrophy (MTA score 2); parietal atrophy (Koedam 2); severe corpus callosum thinning with splenium demyelination (T2/FLAIR hyperintensity, T1 hypointensity)	Severe cognitive disorder requiring permanent care; anxiety and depression prominent
Year 4 (Current)	Severe impairment, no formal testing	Not repeated	Global progression of atrophy; no new lesions	Severe apathy, hypersomnia, total dependence, high wandering risk

↑: above reference range, ↓: below reference range.

## Data Availability

All information included in this article is documented by relevant references.
